# Quality control gone wrong: mitochondria, lysosomal storage disorders and neurodegeneration

**DOI:** 10.1111/bph.12453

**Published:** 2014-03-28

**Authors:** L D Osellame, M R Duchen

**Affiliations:** Department of Cell and Developmental Biology and UCL Consortium for Mitochondrial Research, University College LondonLondon, UK

**Keywords:** mitochondria, neurodegeneration, autophagy, ubiquitin-proteasome system, lysosome, lysosomal storage disorders, Parkinson's disease, Gaucher disease

## Abstract

The eukaryotic cell possesses specialized pathways to turn over and degrade redundant proteins and organelles. Each pathway is unique and responsible for degradation of distinctive cytosolic material. The ubiquitin-proteasome system and autophagy (chaperone-mediated, macro, micro and organelle specific) act synergistically to maintain proteostasis. Defects in this equilibrium can be deleterious at cellular and organism level, giving rise to various disease states. Dysfunction of quality control pathways are implicated in neurodegenerative diseases and appear particularly important in Parkinson's disease and the lysosomal storage disorders. Neurodegeneration resulting from impaired degradation of ubiquitinated proteins and α-synuclein is often accompanied by mitochondrial dysfunction. Mitochondria have evolved to control a diverse number of processes, including cellular energy production, calcium signalling and apoptosis, and like every other organelle within the cell, they must be ‘recycled.’ Failure to do so is potentially lethal as these once indispensible organelles become destructive, leaking reactive oxygen species and activating the intrinsic cell death pathway. This process is paramount in neurons which have an absolute dependence on mitochondrial oxidative phosphorylation as they cannot up-regulate glycolysis. As such, mitochondrial bioenergetic failure can underpin neural death and neurodegenerative disease. In this review, we discuss the links between cellular quality control and neurodegenerative diseases associated with mitochondrial dysfunction, with particular attention to the emerging links between Parkinson's and Gaucher diseases in which defective quality control is a defining factor.

**LINKED ARTICLES:**

This article is part of a themed issue on Mitochondrial Pharmacology: Energy, Injury & Beyond. To view the other articles in this issue visit http://dx.doi.org/10.1111/bph.2014.171.issue-8

## Introduction

Mitochondria are a critical component of the eukaryotic cell, responsible for energy production in the form of ATP, haeme and phospholipid synthesis and calcium buffering (Duchen, 2000). They are also responsible for activation of the intrinsic cell death pathway and can induce apoptosis (Green and Reed, 1998). Mitochondria are organelles enclosed by a double membrane with inner and outer membranes that maintain and separate two distinct aqueous compartments, the inter-membrane space and the matrix. Reflecting their bacterial evolutionary origin the mitochondria have maintained their own genome with 13 respiratory chain proteins, 2 rRNAs and 22 tRNAs encoded (Andersson *et al*., 1998; Levinger *et al*., 2004). Correct mitochondrial function requires a tight coordination between nuclear and mitochondrial encoded genes though various anterograde and retrograde signalling pathways (Ryan and Hoogenraad, 2007).

Neurons have an absolute dependence on mitochondrial oxidative phosphorylation for their ATP supply, with very limited capacity for glycolysis (Herrero-Mendez *et al*., 2009; Bolanos *et al*., 2010). Taking this into account, it is no surprise that the correlation between defects in mitochondrial function and neurodegenerative disorders is relatively high (Schapira *et al*., 1990a,b; Betarbet *et al*., 2000; Cui *et al*., 2006; Lin and Beal, 2006). Many of these reports also specify defects in cellular and mitochondrial quality control (Rubinsztein, 2006; Martinez-Vicente and Cuervo, 2007; Pan *et al*., 2008). Dysfunctional mitochondria can be harmful to the cell as complex I (CI; and to a lesser extent CIII) of the respiratory chain generate damaging reactive oxygen species (ROS; Chance *et al*., 1979; Liu *et al*., 2002). These may in turn cause further damage to the respiratory chain, providing a destructive feedback cycle that can amplify the mitochondrial damage, resulting in neuronal death. Thus, these organelles need to be turned over by the cell's quality control system, and failure of this pathway is strongly associated with neurodegenerative disease (Hara *et al*., 2006; Komatsu *et al*., 2006; Levine and Kroemer, 2008; Narendra *et al*., 2008).

The inner mitochondrial membrane houses the major enzymatic system; the respiratory chain used to transduce oxygen consumption to generate cellular energy in the form of ATP (Mitchell, 1961; Mitchell and Moyle, 1967). The electron transport chain functions by oxidizing NADH and FADH_2_ generated by the citric acid cycle and using these to power the pumping of protons from the matrix into the inter membrane space, generating a potential gradient across the inner membrane (Rich, 2003). It is this potential difference that drives the phosphorylation of ADP to ATP at complex V, the F_1_F_o_-ATP synthase. Mitochondria are the cell's most efficient way of producing energy earning the textbook appellation – ‘the powerhouse of the cell’ (McBride *et al*., 2006; Osellame *et al*., 2012). However, mitochondria are far more complex than a simple cellular battery. In addition to the traditional roles assigned to mitochondria, it is clear they also participate in diverse process such as innate immunity (Seth *et al*., 2005), cardiac and neuronal ischaemia reperfusion (Schinzel *et al*., 2005; Ong *et al*., 2010) and ageing (Ross *et al*., 2013). Hence, defective mitochondrial function will almost inevitably be deleterious to cell and tissue functions threatening the well-being of the entire organism. Impaired mitochondrial function has been associated with various disease states in the CNS, including Leigh syndrome, Freidreich's ataxia and motor neuron, Alzheimer's (AD), Huntington's (HD) and Parkinson's diseases (PDs; Santorelli *et al*., 1993; Mecocci *et al*., 1994; Panov *et al*., 2002; Valente *et al*., 2004). Impaired mitochondrial function is particularly damaging in highly energetic, polarized cells such as neurons (Park *et al*., 2001).

The main clearance pathway for organelle turnover in cells is the autophagic pathway. Central to this pathway is the lysosome, with its low pH and lytic enzymes. Until recently, lysosomes were simply viewed as the organelle responsible for cellular waste disposal. Compelling evidence suggests that the biology of the lysosome extends far beyond the lytic enzymes housed with the lumen (Cesen *et al*., 2012). They are platforms for calcium signalling (Churchill *et al*., 2002; Kilpatrick *et al*., 2013), important in trafficking organelles involved in endocytosis (Chieregatti and Meldolesi, 2005) and involved in nutrient sensing (Sancak *et al*., 2010). The multifactorial role of lysosomes places them at the crossroads of cellular homeostasis (Settembre *et al*., 2013). In autophagy, once the lysosome is fused with the autophagosome, it degrades engulfed organelles and proteins. Alterations in autophagic rate have been implicated in the aforementioned neurodegenerative disorders as well as many lysosomal storage disorders (LSDs; Cuervo *et al*., 2004; Boland *et al*., 2008; Settembre *et al*., 2008a; Osellame *et al*., 2013).

The LSDs are a group of rare inherited metabolic disorders, which result from lysosomal dysfunction that stems from mutations or deficiency of a single lysosomal enzyme. Collectively, LSDs occur at a frequency of ∼1:10 000 with Gaucher disease (GD) as the most prevalent of the group (Meikle *et al*., 1999; Westbroek *et al*., 2011). GD is caused by mutations in the *glucocerebrosidase* (*GBA*) gene and is associated with PD as it appears that similar underlying defects in autophagy and mitochondrial dysfunction may link the neurodegenerative aspect of these two disorders (Tayebi *et al*., 2001; Sun and Grabowski, 2010; Westbroek *et al*., 2011; Osellame *et al*., 2013). Mitochondrial dysfunction has also been associated with other LSDs including mucolipidosis (ML), Batten disease (BD) and multiple sulphatase deficiency (MSD; Jennings *et al*., 2006; Settembre *et al*., 2008a; de Pablo-Latorre *et al*., 2012). It seems that cellular quality control is central to maintaining correct mitochondrial function and protecting from neurodegeneration in numerous disease states (Lee *et al*., 2012).

## Cellular quality control pathways

The eukaryotic cell is equipped with specific machinery to turn over and degrade unwanted/dysfunctional material. These include the ubiquitin-proteasome system (UPS) and the autophagic pathways: chaperone-mediated (CMA), macro, micro as well as organelle specific (pexophagy, reticulophagy, ribophagy and mitophagy; Klionsky and Emr, 2000; Cuervo *et al*., 2004; Dunn *et al*., 2005; Bernales *et al*., 2006; Kim *et al*., 2007; Ron and Walter, 2007; Kraft *et al*., 2008). Each pathway degrades a different type of substrate. The UPS is ultimately responsible for degradation of proteins that are poly-ubiquitinated on lysine 48 and generally have a short half-life (Bence *et al*., 2001). CMA is a highly specialized form of autophagy. In this pathway, chaperones, such as heat shock cognate protein 70 (Hsc70), guide only certain misfolded proteins (such as α-synuclein) to the lysosome (Cuervo *et al*., 2004). Macroautophagy is the bulk cytosolic pathway for relatively non-selective turnover of damaged/dysfunctional organelles and ubiquitinated proteins (modified on lysine 63) with a long half-life (Levine and Klionsky, 2004). Mitophagy is initiated by the damaged mitochondrion itself, which is ultimately degraded by the macroautophagic pathway (Lemasters *et al*., 1998; Narendra *et al*., 2008). While each system possesses qualities that are unique, it is imperative that they act in a cooperative manner to maintain proteostasis.

### Macroautophagy

Macroautophagy, commonly known as autophagy, is the lysosomal-dependent degradation pathway. Autophagy is an essential process and primarily functions to remove damaged and dysfunctional proteins and organelles from the cell.

**Figure 1:**
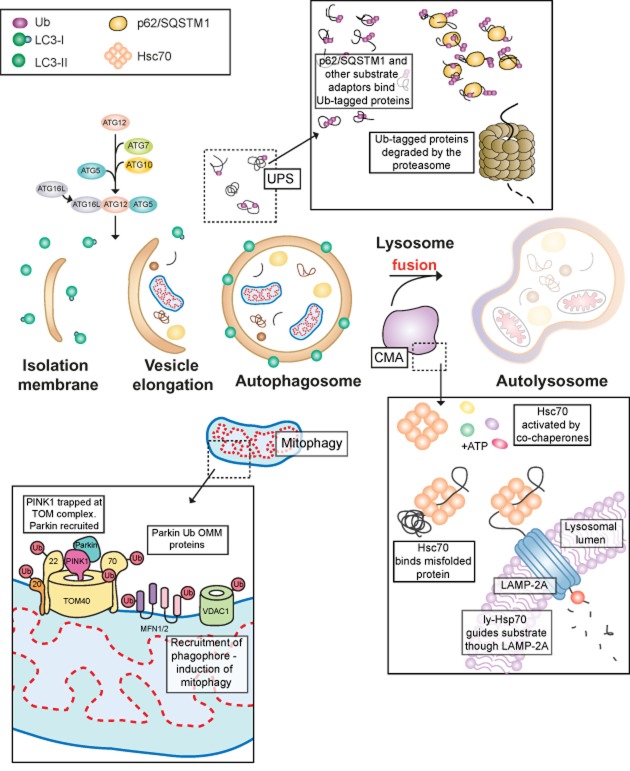
Cellular quality control pathways. Quality control pathways revolve around the autophagy pathway. Expansion of the isolation membrane is initiated by the Atg family of proteins. LC3-I is converted to LC3-II once conjugated to PE on the autophagosome membrane. Once damaged organelles and proteins are engulfed, the autophagosome fuses with the lysosome to form the autolysosome, which facilitates the degradation of the material. The UPS, CMA and mitophagy pathways degrade specific substrates. UPS, poly-ubiquitinated proteins; CMA, specific misfolded proteins; mitophagy, damaged mitochondria.

The initial step of the autophagic pathway – the membrane origins of the autophagosome – remains unclear. There are various reports suggesting that the membrane components could either be generated *de novo* or may arise from other intracellular membrane structures, like that of the endoplasmic reticulum (ER), or more recently reported, the mitochondria (Axe *et al*., 2008; Hailey *et al*., 2010). Initiation is enhanced by activation of Vps34 and its interaction with Beclin1. This step can only proceed once the anti-apoptotic protein Bcl-2 is phosphorylated and thus dissociated from Beclin1, indicating interesting links between the autophagy and apoptosis pathways (Xie and Klionsky, 2007; Funderburk *et al*., 2010). The Atg family of proteins are required for the maturation of the autophagosomal membrane. This stage of the process involves several ubiquitin-like conjugation reactions. The second step of the conjugation system involves Atg8, known in mammalian systems as LC3 (microtubule-associated protein 1A/1B-light chain 3). LC3 is present in two forms depending on the progression of the pathway. In its cytosolic form, it is known as LC3-I; however, when covalently conjugated to phosphatidylethanolamine (PE; in a reaction involving Atg 3 and 7) on the autophagosomal membrane, it is known as LC3-II (Xie and Klionsky, 2007). This form of the protein is present on both the elongating membrane and the newly formed autophagosomes that have engulfed damaged organelles and proteins (Figure 1). It remains on the membrane until fusion with the lysosome. This fusion of the autophagosome and the lysosome results in formation of the lytic organelle, the autolysosome. This step requires functional SNARE [SNAP (soluble NSF attachment protein) receptor)] proteins and has an absolute dependence on normal lysosomal function (Fader *et al*., 2009). The combination of low pH and lytic enzymes from the lysosome and damaged organelles/misfolded proteins from the autophagosome ensure that engulfed material is degraded and the liberated macromolecules recycled for use during cellular starvation. The autophagy pathway is capable of degrading either proteins with long half-lives or organelles. The cytosolic adaptor p62/sequestosome 1 (p62/SQSTM1) traffics poly-ubiquitinated proteins to autophagosomes, possibly via linkage with LC3 in preparation for degradation via autophagy (Bjorkoy *et al*., 2005). Selection of organelles for turnover via autophagy occurs at the organelle level with most possessing specialized, specific pathways to mediate initiation of degradation.

### Mitophagy

The cell possesses organelle-specific turnover pathways in addition to the more general autophagy pathway. Mitochondrial specific degradation, termed mitophagy, requires the coordination of cytosolic factors and signals on the outer mitochondrial membrane (OMM). The process of mitophagy is remarkably specific; by uncoupling a single mitochondrion via photo-irradiation, this and only this mitochondrion will be degraded (Kim and Lemasters, 2011). Further to this, damaging the entire mitochondrial pool using an uncoupler (carbonyl cyanide 3-chlorophenylhydrazone) promotes mitochondrial degradation while leaving other organelles intact (Narendra *et al*., 2008).

The two key regulators of mitophagy are members of the *PARK* family of genes, which associate with familial forms of PD. These include mutations in *PARK6*, which encodes the putative serine/threonine kinase PINK1 (PTEN-induced putative kinase protein 1) and *PARK2* encoding the E3 ubiquitin ligase Parkin (Kitada *et al*., 1998; Valente *et al*., 2004). Parkin mutations account for a high proportion of patients with familial PD, especially those whose onset is considered early (i.e. before 25 years of age; Abbas *et al*., 1999; Lucking *et al*., 2000; Abou-Sleiman *et al*., 2006). PINK1 encodes a 581 amino acid protein, which contains an N-terminal mitochondrial targeting signal and a transmembrane domain that anchors it into the inner mitochondrial membrane (IMM), while the sequence of PINK1 strongly suggests the presence of a C-terminal kinase domain (Silvestri *et al*., 2005; West *et al*., 2005). The structure of Parkin is slightly more complex than that of PINK1. It contains an N-terminal ubiquitin-like domain. The rest of the protein comprises varying RING domains. In the remaining N-terminal domain, Parkin possesses three RING domains and an in-between domain (Deshaies and Joazeiro, 2009). The C-terminus as a whole is termed RING in-between-RING domain (Beasley *et al*., 2007). Most importantly however, this region contains the E3 ubiquitin ligase domain and the recently characterized HECT-like domain. This HECT-like domain is suggested to be responsible for Parkin recruitment to mitochondria and thus activation of mitophagy (Lazarou *et al*., 2013).

PINK1 and Parkin function in the same pathway, with PINK1 acting upstream of Parkin (Figure 1). In viable mitochondria, PINK1 is imported in a membrane potential (ΔΨ_m_)-dependent manner (Jin *et al*., 2010). It normally localizes to the IMM where it is almost immediately cleaved by the rhomboid-like protein PARL (presenilins-associated rhomboid-like protein; Jin *et al*., 2010; Deas *et al*., 2011; Greene *et al*., 2012). Once cleaved, it is degraded by an MG132-sensitive protease (Jin *et al*., 2010). These cleavage steps are critical in normal cell homeostasis as they restrict PINK1 expression. When mitochondria are damaged (and ΔΨ_m_ is dissipated), PINK1 fails to import and accumulates on the OMM where it remains trapped at the TOM complex, recruiting Parkin to the OMM where it exerts its E3 ligase activity (Lazarou *et al*., 2012). Ubiquitination of OMM proteins such as Mfn1/2, VDAC1 and components of the TOM complex (20, 40, 70) ensure that the mitochondrion is truly marked for turnover (Gegg *et al*., 2010; Poole *et al*., 2010; Chan *et al*., 2011). Ubiquitination of VDAC1 is suggested to trigger recruitment of the autophagy adaptor p62/SQSTM1, in a role similar to that it performs in the cytosol, where it essentially ‘chaperones’ ubiquitinated proteins to the proteasome, activating recruitment and conjugation of the phagophore, and ensuring encapsulation and eventual turnover of the damaged mitochondrion (Geisler *et al*., 2010).

The manner in which Parkin ubiquitinates OMM proteins has recently been identified. Although there do not appear to be sequence motifs common to proteins ubiquitinated by Parkin, it seems that the rationale for the modification of proteins is actually very simple. Sole recruitment of Parkin to the outer membrane and proximity to target proteins may be all that is required for ubiquitination (Sarraf *et al*., 2013). Supporting this, it seems that only the cytoplasmic face of the outer membrane proteins are modified (Sarraf *et al*., 2013). Perhaps Parkin ubiquitination is less selective than first thought? Recently, an alternative Parkin receptor has been proposed, adding another piece to an already complex puzzle. Mitofusin 2 (Mfn2; an OMM GTPase primarily responsible for mitochondrial fusion), is phosphorylated by PINK1 and has been shown to be indispensible for depolarization-induced Parkin translocation to mitochondria in cardiomyocytes (Chen and Dorn, 2013). However, there is most likely some redundancy in the recruitment system, as Parkin still translocates to the OMM in Mfn2-depleted embryonic fibroblasts (Chan *et al*., 2011). Whether this specific mechanism is of primary importance in the heart and a secondary mechanism in other tissues remains to be seen.

Although a complex multi-step process, regulation of mitophagy under the PINK1/Parkin (Mfn2?) system does seem logical. However some doubts remain as to the physiological relevance of this pathway. Although well characterized, experimental demonstration of this model pathway relies on complete depolarization of the ΔΨ_m_ with prolonged exposure to high doses of uncoupler. Whether an equivalent process actually occurs *in vivo* and in disease states where mitophagy is defective (i.e. PD and some LSDs) is not clear.

### Chaperone-mediated autophagy (CMA)

CMA is one of the lysosomal pathways of proteolysis. It is markedly different to conventional autophagy as no vesicular transport is involved; instead, cytosolic proteins are recognized and delivered to the lysosome by chaperones in a molecule-by-molecule-dependent manner (Dice, 2007). In this fashion, the mechanism of CMA is similar to that of protein import into the mitochondria. Cytosolic proteins with the KFERQ-like motif are recognized by the cognate receptor chaperone Hsc70 (Chiang *et al*., 1989; Terlecky *et al*., 1992). This motif and slight modifications of it are present in 30% of cytoplasmic proteins, thus CMA accounts for a significant portion of the turnover of misfolded/damaged proteins (Chiang and Dice, 1988). Included in this group are mutant huntingtin and α-synuclein, associated with HD and PD respectively. While it has been proposed that both autophagy and the UPS degrade α-synuclein, impaired degradation of mutant α-synuclein by CMA has been generally implicated in neurodegeneration (McNaught *et al*., 2001; Cuervo *et al*., 2004; Ebrahimi-Fakhari *et al*., 2011).

Under normal circumstances CMA is induced under starvation conditions with heat shock protein 40 (Hsp40) stimulating Hsc70 activity, which then binds substrate proteins in an ATP-dependent manner. Hsc70 (along with co-chaperones hip, hop, bag-1, Hsp40 and 70) transport the cytosolic protein to the membrane of the lysosome where they bind to lysosome-associated membrane protein 2A (LAMP-2A; Agarraberes and Dice, 2001; Figure 1). Like mitochondrial protein import, these proteins must be unfolded prior to transport into the lysosomal lumen and the chaperones are vital for this process. The binding of Hsc70 and the substrate to LAMP-2A monomers, triggers the assembly of LAMP-2A multimers to form a translocation complex though which the substrate can pass, although in an unfolded state. Lysosomal heat shock cognate 70 (Ly-Hsc70), the lysosomal form of the chaperone, is required to ‘pull’ the translocated protein through the membrane receptor LAMP-2A (Agarraberes *et al*., 1997). Post-translocation, these proteins are rapidly degraded by lysosomal hydrolases. Binding and translocation to and across LAMP-2A appear to be limiting factors in the causation of PD; α-synuclein mutations in the KFERQ-like motif have been shown to bind to LAMP-2A but fail to be translocated, leaving them in an aggregated/misfolded state on the lysosome (Cuervo *et al*., 2004). As CMA degradation occurs in a molecule-by-molecule fashion, binding of mutant α-synuclein renders this process inactive as uptake/binding and degradation of other CMA substrates is inhibited leading to impaired proteostasis, which probably contributes to the pathophysiology of PD and synucleinopathies.

### Ubiquitin-proteasome system

The UPS prototypically recognizes specific protein substrates that have been covalently modified by the addition of ubiquitin (Ub), a small 76 amino acid polypeptide with poly-ubiquitin marking the substrate for transportation to the proteasome (Korolchuk *et al*., 2010). The UPS is responsible for turnover of ubiquitinated misfolded/damaged proteins with a short half-life. This is a highly catabolic process that requires energy in the form of ATP, primarily generated from mitochondria. The UPS is a highly regulated process under the control of E1 (ubiquitin-activating enzyme), E2 (Ub conjugating enzyme) and E3 (ubiquitin ligase) enzymes, each playing a specific role in post-translational modification of target proteins (Deshaies and Joazeiro, 2009). The E1 and E2 class of enzymes activate the ubiquitin in an ATP-dependent process while the E3 ligase performs the final step in transferring the activated ubiquitin to the ε-amino group of the lysine residue in the target protein (Hershko *et al*., 1983; Pickart and Eddins, 2004). Degradation of the targeted protein by the UPS requires poly-ubiquitination at lysine 48 (Rodrigo-Brenni *et al*., 2010). These proteins are transported by the cytosolic adaptor p62/SQSTM1 and various ubiquitin receptor proteins, which function as ubiquitin-binding scaffold proteins, binding aggregates in the cytosol (Elsasser and Finley, 2005). As a key component of the ubiquitin system, p62/SQSTM1 is a vital link between the UPS and autophagy, and is itself ultimately degraded by autophagy (Seibenhener *et al*., 2004; Korolchuk *et al*., 2009). It is a common component of protein aggregates and Lewy bodies (LB) found in PD, mutant huntingtin aggregates in HD and neurofibrillary tangles in AD (Kuusisto *et al*., 2001; 2002[Bibr b88],[Bibr b87]; Nagaoka *et al*., 2004; Bjorkoy *et al*., 2005).

The proteasome is a barrel-shaped proteolytic organelle expressed throughout the cell. It consists of a central 20S subunit and two 19S ‘lid’ units. The 20S subunit is considered the proteolytic core of the complex, with the 19S units serving as the protein-binding components (Dahlmann *et al*., 1986; Lowe *et al*., 1995). The proteasome has relatively broad activity divided into three main classes – chymotrypsin-like, trypsin-like and peptidlyglutamyl–peptide hydrolysing (Heinemeyer *et al*., 1997). The catalytic pore of the proteasome is roughly 53 angstroms wide, although the entry point can be as narrow as 13 angstroms, suggesting that the ubiquitinated proteins must be at least partially unfolded to enter the catalytic core (Nandi *et al*., 2006). This partial unfolding appears to be one of the factors associated with the accumulation of ubiquitinated proteins in neurodegenerative diseases; highly aggregated proteins are poor proteasomal substrates as they cannot be easily unfolded. As such, an impaired UPS is often suggested as an underlying molecular mechanism in disease states such as PD, AD and HD.

## Mitochondrial dysfunction in LSDs

Lysosomes were first described by Christian de Duve in 1949 and the name is derived from the Greek word *lysis* (to separate) and *soma* (body) (De Duve *et al*., 1955). They range in size from 0.1–1.2 μm with an acidic luminal pH of around 4.8, which is essential for lysosomal function (Mullins and Bonifacino, 2001). They are membrane-bound organelles and contain at least seven integral membrane proteins and about 50–60 soluble hydrolases (Futerman and van Meer, 2004). Mutations in genes that encode these proteins can cause LSDs, some of which are shown in Table 1. More than 40 LSDs have been described and these are classified and grouped according to the nature of the accumulated substrate. Most LSDs are inherited in an autosomal recessive manner and present as infantile forms of the disease. However, Fabry disease and Hunter syndrome (MPS II) are X-linked and recessively inherited (Shachar *et al*., 2011). Altered mitochondrial function has been reported in many LSDs, namely, MSD (Settembre *et al*., 2008a; de Pablo-Latorre *et al*., 2012), ML II, ML III (Otomo *et al*., 2009), ML IV (Jennings *et al*., 2006), GM1-gangliosidosis (GM1; Takamura *et al*., 2008; Sano *et al*., 2009), neuronal ceroid-lipofuscinoses or BD (NCL3; Cao *et al*., 2006) and GD (Sun and Grabowski, 2010; Cleeter *et al*., 2013; Osellame *et al*., 2013).

**Table 1:** Lysosomal storage disorders

Disease	Gene (protein)	Accumulated substrate	CNS affected	QC affected	Mitochondria affected
Gaucher	GBA (GBA/GCase)	GBA	+	+	+
Nieman-Pick type C	NPC1/2 (Neiman-Pick C 1/2)	Sphingolipids and cholesterol	+	+	−
Mucopolysaccharidosis					
Type II (Hunter syndrome)	I2S (iduronate-2-sulphatase)	Heparan sulphate and dermatan sulphate	+	+	+
Type IIIA (Sanfilippo syndrome)	SGSH (heparan N-sulphatase)	Glycosaminoglycan heparan sulphate	+	+	+
Type IIIB (Sanfilippo syndrome)	NAGLU (N-acetyl-α-D glucosaminidase)	Heparan sulphate	+	−	+
Multiple sulphatase deficiency	SUMF1 (sulphatase-modifying factor-1)	Sulphatides, sulphated glycosaminoglycans, sphingolipids and steroid sulphates	+	+	+
Fabry	GLA (α-galactosidase)	Globotriasylceremide	−	+	+
Tay-Sachs (GM2 gangliosidosis/hexosaminidase A deficiency	HEXB (β-hexosaminidase)	GM2 ganglioside	+	+	−
Lipofuscinosis (NCLs)					
Type I	CLN1 (palmitoyl protein thioesterase)	Lipodated thioesters and lipofusin	+	−	−
Type III (Batten)	CLN3 (ceroid-lipofuscinosis 3/battenin)	Subunit c of the mitochondrial ATP synthase/complex V	+	+	+
Pompe	GAA (acid-α-glucosidase)	Glycogen	+	+	−
Mucolipidosis					
Type II-II	GNPTAB (N-acetylglucosamine-1-phosphotransferase)	N-linked glycoproteins	+	−	+
Type IV	MCOLN1 (mucolipin 1)	Phosphatidylcholine, lysophosphatidylcholine, phosphatidylethanolamine	+	+	+

A selection of LSDs that harbour CNS abnormalities, quality control (QC) defects and/or mitochondrial dysfunction.

### Multiple sulphatase deficiency

MSD, also known as Austin disease and mucosulfatidosis, is a rare LSD caused by mutations in the *SUMF1* gene, resulting in an accumulation of sulphatides, sulphated glycosaminoglycans, sphingolipids and steroid sulphates (Austin, 1973; Cosma *et al*., 2003; Dierks *et al*., 2003). Affected individuals present with neurological deterioration, ichthyosis, skeletal anomalies and organomegaly. There are three types of MSD, differentiated according to age of onset – neonatal, late infantile and juvenile, with neonatal being the most severe and results in death in the first year of life (Dierks *et al*., 2003).

In murine models of MSD, defects in both autophagy and mitophagy have been observed (Settembre *et al*., 2008a; de Pablo-Latorre *et al*., 2012). Mitochondria in these models were found to be fragmented with a reduced ΔΨ_m_. Originally, it was proposed that the inability to turn over these dysfunctional mitochondria was due to ineffective lysosome-autophagosome fusion, as autophagosome number was increased (Settembre *et al*., 2008a,b). However, it appears that impaired Parkin-mediated ubiquitination of the OMM may contribute to defective proteostasis (de Pablo-Latorre *et al*., 2012). As well as a decreased ΔΨ_m_, the mitochondrial network was fragmented and ATP production was reduced, indicating a generalized mitochondrial dysfunction. Parkin failed to translocate to the OMM, and OMM proteins were only partially ubiquitinated; thus, the mitochondria were not tagged for degradation (de Pablo-Latorre *et al*., 2012). However, this study by de Pablo-Latorre and colleagues described liver mitochondria; the same ‘mitochondrial priming’ (i.e. marking the organelle for turnover) was not seen in the brain. Given the progressive degenerative nature of MSD, it is unclear whether there are similar mitophagy defects in the brain that may have been masked by the nature of the experimental material (whole brain) in this study. Perhaps this again suggests (like Mfn2 as a potential redundant receptor in the heart) that the regulation of the mitophagy pathway is tissue specific.

### Mucolipidosis (ML)

ML is a collective name for the group of autosomal recessive diseases in which the accumulated substrate is phospholipid (Mancini *et al*., 2000; Mach, 2002). There are four types of ML, types I–III involve the mis-targeting of lysosomal lipid hydrolases, resulting in inefficient processing of endocytosed lipids (Bach and Desnick, 1988; Bargal and Bach, 1989; Slaugenhaupt, 2002). Type IV (ML IV), however, differs slightly as it is linked to mutations in the proposed ion-channel, mucolipin 1 (MCOLN1), a protein suggested to play a role in lysosomal/endosome function (Bargal and Bach, 1989; Fares and Greenwald, 2001; LaPlante *et al*., 2004). Interestingly, ML IV is often misdiagnosed as cerebral palsy; thus, the reported incidence of 1:40 000 is thought to be conservative (Altarescu *et al*., 2002). The carrier rate among the Ashkenazi population is highly elevated in comparison to the general population, with two founder mutations (c416-2 A > G and C.1_788del) giving rise to a carrier rate of 1:90–1:100 (Bargal *et al*., 2001; Bach *et al*., 2005).

Fibroblasts from ML IV harbour dysfunctional mitochondria. Mitochondria in type IV (and II, III) were fragmented with a reduced ability to buffer Ca^2+^ (Jennings *et al*., 2006). This is problematic for neuronal cells as reduced calcium uptake can render them particularly vulnerable to calcium-mediated apoptosis (Duchen, 1999; Kruman and Mattson, 1999). And as such, ML IV cells were more susceptible to stress-induced cell death (caspase 8 dependent; Jennings *et al*., 2006). This suggests that impaired lysosomal function affects the autophagic turnover of dysfunctional mitochondria, which therefore show increased susceptibility to stress-induced apoptosis.

### GM1-gangliosidosis

GM1-gangliosidosis is a rare inherited disorder that is generally characterized by progressive neurodegeneration (Brunetti-Pierri and Scaglia, 2008). GM1-gangliosidoses are caused by recessive mutants in the β-galactosidase gene (Suzuki and Oshima, 1993). The protein β-galactosidase is a lysosomal hydrolase that hydrolyses the terminal galactosyl residues from GM1-ganglioside, glycosaminoglycans and glycoproteins (Okada and O'Brien, 1968). There are three clinical subtypes of GM1-gangliosidosis, which are generally classified by age of onset. Type I (infantile) presents most frequently at birth or in early infancy with neurolipidosis (neurodegeneration with cherry-red spots in the eye; Brunetti-Pierri and Scaglia, 2008). Type II presents with a viable clinical phenotype including neurodegeneration, ataxia and seizures. Type II (adult) is classified as late age of onset and presents with progressive dementia, Parkinsonism and dystonia (Suzuki, 1991; Roze *et al*., 2005). Only patients with type III GM1-gangliosidosis survive to adulthood.

The severity of the disease is related to the residual activity of the mutant β-galactosidase enzyme with the most severe mutations resulting in little to no enzyme found at lysosomes (Suzuki *et al*., 1978). Using knockout mouse models of β-galactosidase, it has been suggested that enhanced autophagy and mitochondrial dysfunction may be responsible for some aspects of neurodegeneration in GM1-gangliosidosis (Takamura *et al*., 2008; Sano *et al*., 2009). Both LC3-II and Beclin1 levels were enhanced in the brain in addition to activation of Akt-mammalian target of rapamycin (mTOR) and Erk signalling pathways. At the organelle level, increased activity of cytochrome *c* oxidase (CIV) was observed in astrocytes as well as abnormal mitochondrial morphology and a decrease in ΔΨ_m_ (Takamura *et al*., 2008). Cellular dysfunction was attenuated by inhibiting autophagy (with 3-methyl adenine), suggesting that overactivation of autophagy may play a role in the pathophysiology of GM1-gangliosidosis (Takamura *et al*., 2008). Deregulated calcium signalling has also been postulated to play a role in GM1-gangliosidosis. As well as accumulating in lysosomes, GM1-ganglioside has been shown to associate with glycophospholipid-enhanced microdomains of mitochondrial associated ER membranes (Sano *et al*., 2009). Here, it is proposed to interact with the phosphorylated form of the inositol triphosphate receptor, affecting the activity of the receptor and resulting in perturbations in calcium uptake by mitochondria, leading to organelle dysfunction (Sano *et al*., 2009).

### Batten disease (NCL type III)

Neuronal ceroid lipofuscinosis (NCL) or BD are recessively inherited neurodegenerative disorders, characterized by lysosomal accumulation of subunit c of the ATP synthase within neurons (Palmer *et al*., 1992). They are the leading cause of neurodegeneration among children and are always fatal (Wisniewski *et al*., 2001). Although rare, BD is found in all populations, it is however more prevalent in Finland where it is thought to occur at a frequency of 1:10 000 (Santavuori, 1988). A founder mutation in the gene CLN3 (also known as battenin) results in a 1.02 kb deletion of genomic DNA (gDNA), resulting in deletions of exons 7 and 8 and the flanking intronic gDNA. This causes multiple copies of stable mutant CLN3 mRNA isoforms, all of which appear to be non-functional. The wild-type CLN3 protein, localizes to the lysosome/endosome. The protein is thought to be responsible for assisting vesicular trafficking and in regulating pH and amino acid transport. The accumulated material, subunit c of the ATPase, normally resides within the IMM as a component of the respiratory chain enzyme, complex V (CV; ATP synthase), where it is responsible for the conversion of inorganic phosphate and ADP to ATP, the cellular unit of energy (Rich, 2003). Although the underlying mechanism of the disease has yet to be elucidated, alterations in mitochondrial function have been reported. Indeed, some reports regarding BD suggest that mitochondrial metabolism is defective. Reductions in β-oxidation of palmitate have been shown in patient fibroblasts, although it seems that respiratory chain function in these cells is normal, suggesting that the selective turnover of components of the mitochondria (namely, IMM) is defective (Hall *et al*., 1991; Palmer *et al*., 1992; Dawson *et al*., 1996). A mouse knock-in model of the exon7/8 CLN3/battenin deletion reveals defects in the autophagy pathway, stemming from defects in autophagosome maturation (Cao *et al*., 2006). Surprisingly, both subunit c and CLN3/battenin were found to be enriched in the membrane of these delayed autophagomes, hinting that the mitochondria may well be involved in the origins of autophagosome formation (Cao *et al*., 2006). The delayed progression of the autophagic pathway has implications for organelle turnover in this disease and although accumulation of subunit c of mitochondrial CV is specifically associated with BD, it appears that it may be a secondary effect due to impaired autophagy in this disease state.

### Gaucher disease (GD)

GD is caused by recessive mutations in the *GBA* gene. The *GBA* gene is located on chromosome 1q21 and contains 11 exons and 10 introns spanning a 7.6 Kb region. The functional gene shares 96% exonic homology with a non-processed pseudogene (16 Kb upstream) (Hruska *et al*., 2008). It is the presence of this highly homologous pseudogene along with six other genes in the region (including Metaxin1) that can result in misalignments and chromosomal rearrangements which explain the relatively high amount of recombinant alleles detected in GD (Hruska *et al*., 2008).

The protein product of *GBA* is glucerebrosidase (GCase) and is responsible for the conversion of its substrate glucocerebroside to glucose and ceramide (Brady *et al*., 1965). In the disease state, deficiency of the enzyme results in accumulation of toxic substrate within the lysosome. Although classified as a pan-ethnic disorder, prevalence varies depending on population bias. In the general population occurrence is estimated as 1:40 000; however, among Ashkenazi Jews, the prevalence is thought to be as high as 1:900 (Zimran *et al*., 1991; Aharon-Peretz *et al*., 2004). Classed as an in-born error of metabolism, GD can be further classified into three types based on age of onset and neurological involvement (Grabowski, 2008). Type I (OMIM#230800) is the most common and is defined by a lack of neurological features. Type II (OMIM#230900) presents very early in life and is classed as acute infantile neuronopathic GD. Type III (OMIM#2301000) also presents with neurological involvement but has a more chronic presentation. In many cases of GD, the relationship between genotype and phenotype is obscure and often does not correlate with enzyme activity, making it very difficult to classify definitively (Grabowski, 2008). In addition, all types of GD present very differently in the clinic and it has been reported than even affected siblings with the same type of GD will present differently. Type 1, the mildest form, presents with skeletal abnormalities and enlarged liver (hepatomegaly) and/or spleen (splenomegaly) among others (Grabowski, 2008). Type II presents with hepatosplenomegaly, joint pain and CNS involvement – seizures and mental retardation (Grabowski, 2008). The visceral symptoms of type I GD are currently treatable with enzyme replacement therapy such as imiglucerase and velaglucerase; however, these enzymes do not cross the blood–brain barrier and are thus not effective for types II and III (Zimran *et al*., 2013).

The molecular mechanism underlying the pathology of GD is poorly defined. Although the primary defect appears to be lysosomal, the effects at cellular and organism level are only beginning to be considered as the links between GD and PD come to light (Sidransky, 2005; Dehay *et al*., 2010; Mazzulli *et al*., 2011; Sidransky and Lopez, 2012; Cleeter *et al*., 2013; Dehay *et al*., 2013; Osellame *et al*., 2013).

### Links between GD and PD

PD is a progressive age-related neurodegenerative disorder affecting 1% of the world's population over the age of 65 (DePaolo *et al*., 2009). Physical symptoms include bradykinesia, tremors at rest, postural instability and rigidity. The neuropathology is marked by the degeneration of dopaminergic neurons in the substantia nigra pars compacta (SN) and accumulation of α-synuclein positive LB within neurites.

Although the molecular mechanism of PD remains unclear, much of the current understanding has been gained by identification of distinct loci at which pathogenic mutations are associated with Parkinsonism (Table 2). Of these, mutations in *GBA* are numerically identified as the highest known genetic risk factor for PD (Neudorfer *et al*., 1996; Neumann *et al*., 2009; Lesage *et al*., 2011). Parkinsonism is an established feature of GD and the pathological and neurological symptoms displayed by GD patients share many features of PD. Several large multi-centre genome wide association studies have reported high incidences of *GBA* mutations in sporadic PD (Sidransky, 2005; Mata *et al*., 2008; Mitsui *et al*., 2009; Sidransky *et al*., 2009a,b; Sidransky and Lopez, 2012). *Post-mortem* analysis of PD patient brains with *GBA* mutations revealed extensive GCase deficiency in the brain with the SN most affected (Gegg *et al*., 2012). In addition, involvement of an α-synuclein feedback loop in GD and synucleinopathies suggests that accumulating oligomeric α-synuclein inhibits the activity of endogenous wild-type GCase in idiopathic PD brains, contributing to the progression of sporadic PD and synucleinopathies (Mazzulli *et al*., 2011; Gegg *et al*., 2012; Manzoni and Lewis, 2013). This mechanism appears to be similar to that of a self-propagating prion-like disease; where α-synuclein and GCase provide the basis for a destructive feedback loop. Pharmacological inhibition of GCase by a specific inhibitor (conduritol-β-epoxide) mimics the *in vivo* disease state. Chemical inhibition in the neuronal-like SH-SY5Y cells also led to α-synuclein accumulation. The mitochondrial pool is also affected resulting in decreased ADP phosphorylation and reduced ΔΨ_m_ (Cleeter *et al*., 2013). These studies suggest that decreased GCase activity, similar to that observed with pathogenic mutations of GD, is the prime cause of the pathology associated with GD. Close to 300 unique mutations of *GBA* have been reported to be associated with GD, two of the most prevalent being L444P and N370S, with the latter found in >98% of Jewish patients and about half of non-Jewish patients (Beutler and Gelbart, 1993; Beutler *et al*., 1993; Hruska *et al*., 2008). These and other mutations have been reported to cause defects of GCase trafficking, with the mutated protein trapped in the ER, triggering the unfolded protein response and undergoing ER-associated degradation via the proteasome (Aharon-Peretz *et al*., 2004; Wong *et al*., 2004; Gegg *et al*., 2012). As a result, very little mutated GCase may actually be localized to the lysosome. Either theory is enticing (self-propagating feedback loop or ER-mediated degradation) as they present potential therapeutic options for increasing GCase targeting to the lysosome.

**Table 2:** Parkinson's disease associated genes

Locus	Gene	Function	Clinical presentation
*PARK1/4*	α-synuclein	Suggested involvement in synaptic function	Parkinsonism, dementia with LB
*PARK2*	Parkin	E3 ubiquitin ligase	Early onset Parkinsonism
*PARK5*	UCHL1	Deubiquitinating enzyme	Late onset Parkinsonism
*PARK6*	PINK1	Involved in sensing mitochondrial oxidative/stress. Putative kinase activity	Early onset Parkinsonism, very rarely associated with LB
*PARK7*	DJ-1	Involved in oxidative stress response	Early onset Parkinsonism
*PARK8*	LRRK2	PK	Late onset Parkinsonism, with LB
*PARK9*	ATP13A2	Encodes a lysosomal P-type ATPase. Exact function unknown	Early onset Parkinsonism with Kufer-Rakeb syndrome
*PARK13*	Htra2/Omi	Serine protease	Early onset Parkinsonism
*PARK15*	FBXO7	F-box protein, component of ubiquitin ligase complex	Early onset Parkinsonism with Pallido-pyramidal syndrome
*GAUCHER*	GBA	Lysosomal enzyme involved in glycolipid metabolism	GD, late onset Parkinsonism with LB

ATP13A2, ATPase type 13A2; FBXO7, F-box protein 7; Htra2/Omi, high-temperature requirement protein A2/serine protease 25; LRRK2, leucine-rich repeat kinase 2; UCHL1, ubiquitin carboxyl-terminal hydrolase L1.

Abundantly clear however is the absolute requirement for active GCase in clearance of proteins such as α-synuclein and maintaining proteostasis. Complete depletion of GCase in a type II neuronopathic GD mouse model results in a global decrease in quality control mechanisms in neurons (not just CMA of α-synuclein). In this system, knockout (KO) of GCase in primary neurons and astrocytes caused defects in the autophagy pathway with reductions in LC3-I/II and Atg5/12 levels, suggesting the presence of a negative feedback loop upstream of lysosomal involvement (Osellame *et al*., 2013). Defective autophagy impinges on the UPS with increases in ubiquitinated proteins, p62/SQSTM1 aggregates as well as α-synuclein deposits in the midbrain. Defective mitochondrial quality control is associated with mutations in the *PARK* family of genes and can cause familial PD, yet very little is known about the mitochondrial involvement in GD. It was shown that as a result of defective quality control mechanisms dysfunctional mitochondria in type II GD do not recruit the E3 ubiquitin ligase Parkin, and are not flagged for turnover and accumulate. GCase KO cells showed reduced mitochondrial CI, CII/III activity and reduced ΔΨ_m_. Similar to that of PINK1 knock-down in neurons, mitochondrial membrane potential is maintained by ATP hydrolysis by the F1Fo-ATP synthase in type II GD neurons and astrocytes – the mitochondria are no longer serving as sources of energy but rather as ATP consumers (Gandhi *et al*., 2009; Osellame *et al*., 2013). This coincides with mitochondrial fragmentation due to increased OPA1 processing. Interestingly, loss of ΔΨ_m_ can be attenuated by the mitochondrial targeted antioxidant MitoQ_10_ (a ROS scavenger) suggesting that damage is mediated by ROS generation from CI (James *et al*., 2004; Murphy and Smith, 2007; Osellame *et al*., 2013). Treatment with MitoQ_10_ did not restore defects in the autophagy pathway, confirming that the primary lysosomal defect affects cellular quality control (Osellame *et al*., 2013). Taken together, these findings suggest that cellular dysfunction observed in GD, like that of PD, is a consequence of defects in autophagy/mitophagy pathways, resulting in failed clearance of damaged mitochondria.

## The bigger picture: mechanistic links between LSDs and neurodegeneration

There appears to be a strong clinical association between these two classes of disease. As the lysosome is central to the autophagy process, it is not unreasonable to assume this pathway is integral in protecting against neurodegeneration (Xilouri and Stefanis, 2011). Many of the products accumulated in LSDs coincide with α-synuclein aggregation and the presence of LB, thus it has been suggested that LSDs could be classified as ‘autophagy disorders’ (Settembre *et al*., 2008b). This may be the case with GD associated with PD. Accumulation of α-synuclein and dysfunctional mitochondria, which under normal cell homeostasis, should be turned over by CMA/autophagy and mitophagy/autophagy, respectively, suggests this may be the case. In addition, the UPS has also been implicated in both disease states (McNaught *et al*., 2001; Ross and Pickart, 2004; Ron and Horowitz, 2005). Taken together, global defects in all aspects of cellular quality control link the neurodegeneration seen in both PD and LSDs. Although an overly simplistic view, up-regulation of autophagy in diseases such PD and GD may alleviate some of the clinical and pathological symptoms (Rubinsztein *et al*., 2007; Fleming *et al*., 2011; Santos *et al*., 2011). However, pharmacological treatment with drugs such as rapamycin, which inhibits mTOR and thus induces autophagy, will result in undesirable off-target effects especially when required for chronic treatment (Rubinsztein *et al*., 2007). Lentiviral delivery of Beclin-1 may be a more effective option to ameliorate autophagy and has been shown to reduce α-synuclein accumulation *in vivo* (Spencer *et al*., 2009). Another option for monogenic disorders may be intervention by gene therapy; reintroducing a functional form of the gene via viral transduction (Enquist *et al*., 2006; Rahim *et al*., 2009). As many of the diseases discussed here cause rather severe neurodegeneration in infancy, both pharmacological and gene therapy treatments would need to be applied in an early therapeutic window (Waddington *et al*., 2005). As the intricate nature of quality control pathways, the downstream effect on mitochondria and the neurodegeneration that follows is beginning to come to light, the interplay between these may present excellent therapeutic targets for clinical intervention.

## Abbreviations

CI: complex I
CMA: chaperone-mediated autophagy
CV: complex V
GBA: glucocerebrosidase
GD: Gaucher disease
Hsc70: heat shock cognate 70
Hsp70: heat shock protein 70
IMM: inner mitochondrial membrane
LAMP-2A: lysosome-associated membrane protein 2A
LC3: microtubule-associated protein 1A/1B-light chain 3
LSDs: lysosomal storage disorders
OMM: outer mitochondrial membrane
p62/SQSTM1: p62/sequestosome 1
PD: Parkinson's disease
ROS: reactive oxygen species
UPS: ubiquitin-proteasome system
